# Association of *ABHD12* Variants with the Spectrum of PHARC Syndrome Phenotypes

**DOI:** 10.3390/ijms27167506

**Published:** 2026-08-21

**Authors:** Sara Romero-Vázquez, Cécile Méjécase, Ana Catalina Rodriguez-Martinez, Nicola Cronbach, Mariya Moosajee

**Affiliations:** 1 UCL Institute of Ophthalmology, London EC1V 9EL, UK; s.vazquez@ucl.ac.uk (S.R.-V.); c.mejecase@ucl.ac.uk (C.M.); ana.martinez.22@ucl.ac.uk (A.C.R.-M.); nicola.cronbach.23@ucl.ac.uk (N.C.); 2 The Francis Crick Institute, London NW1 1AT, UK; 3 Moorfields Eye Hospital NHS Foundation Trust, London EC1V 2PD, UK

**Keywords:** *ABHD12*, ataxia, cataracts, hearing loss, PHARC, polyneuropathy, retinitis pigmentosa, VLC-lysophosphatidylserine

## Abstract

PHARC (polyneuropathy, hearing loss, ataxia, retinitis pigmentosa and cataracts) syndrome is a rare neurodegenerative inherited disorder characterized by a spectrum of these clinical phenotypes. Due to the considerable heterogeneity in the age of onset of the different clinical features and the similarities with other conditions, it is often misdiagnosed. Variants in the *ABHD12* gene have been identified as a genetic cause of PHARC syndrome and are predicted to result in loss-of-function or severely reduced protein synthesis. ABHD12 (α/β-hydrolase domain-containing protein 12) is responsible for the breakdown of very-long-chain lysophosphatidylserines (VLC-lyso-PS). When ABHD12 is dysfunctional, VLC-lyso-PS accumulates in the brain, producing inflammation. In this study, the clinical phenotypes of five patients with molecularly confirmed *ABHD12* pathogenic variants from Moorfields Eye Hospital together with those of 64 patients reported in the literature were analyzed to investigate genotype–phenotype correlations. We report a novel variant c.985del p.(His329Thrfs*57) associated with PHARC syndrome. Moreover, patients with two null variants in *ABHD12* manifest more of the clinical features of PHARC syndrome than those with at least one missense variant. Our study shows the importance of a complete clinical and genetic diagnosis to enable accurate diagnosis and genetic counselling for affected individuals.

## 1. Introduction

PHARC syndrome encompasses demyelinating sensorimotor polyneuropathy, hearing loss, ataxia, retinitis pigmentosa (RP) and early-onset cataracts (OMIM 612674). Historically underdiagnosed due to its resemblance to Charcot–Marie–Tooth disease (MIM 118220), Usher syndrome (MIM 276900), and Refsum disease (MIM 266500) and because of the lack of widespread genetic testing [1,2,3,4]. This autosomal-recessive, slowly progressive neurodegenerative disorder is caused by biallelic variants in *ABHD12* [1]. Located on chromosome 20p11, the *ABHD12* gene produces two alternative splice isoforms: *ABHD12-a* (NM_001042472.3, canonical transcript) and *ABHD12-b* (NM_015600.5). They only differ in the last exon, producing a 398-residue (ABHD12-a) and a 404-residue (ABHD12-b) protein.

PHARC syndrome was first described in 2009 in three patients from a consanguineous Norwegian family exhibiting: sensory and motor neuropathy, sensorineural hearing loss, ataxia, retinal disease and cataract. Their clinical features were reminiscent of Refsum-like disease except for the absence of peroxisomal dysfunction. The disease was mapped to a 16 Mb region on chromosome 20 (20p11.21-q12); however, no mutation was found in the 23 genes sequenced [5]. The inclusion of 19 more patients from 11 families the year after allowed the refinement of the candidate region and mutations in the *ABHD12* gene were identified as the cause of PHARC syndrome [1].

ABHD12 (α/β-hydrolase domain-containing protein 12) is an integral membrane enzyme member of the serine hydrolases superfamily that degrades lysophosphatidyl serine (lyso-PS) in the endoplasmic reticulum (ER) [6,7,8]. ABHD12 is anchored to the ER through an N-terminal transmembrane helix, and the C-terminal site is predictively orientated towards the ER lumen [9,10,11,12]. In these loop regions lies the highly conserved catalytic triad, with a nucleophilic residue, an acidic residue and a histidine (Ser-Asp-His in ABHD12). A highly conserved acyltransferase activity motif (His-XXXX-Asp) can also be found [13]. The ABHD12 protein is ubiquitously expressed in mammals, with its highest levels observed in brain tissue, particularly in microglial cells [14].

**Figure 1:**
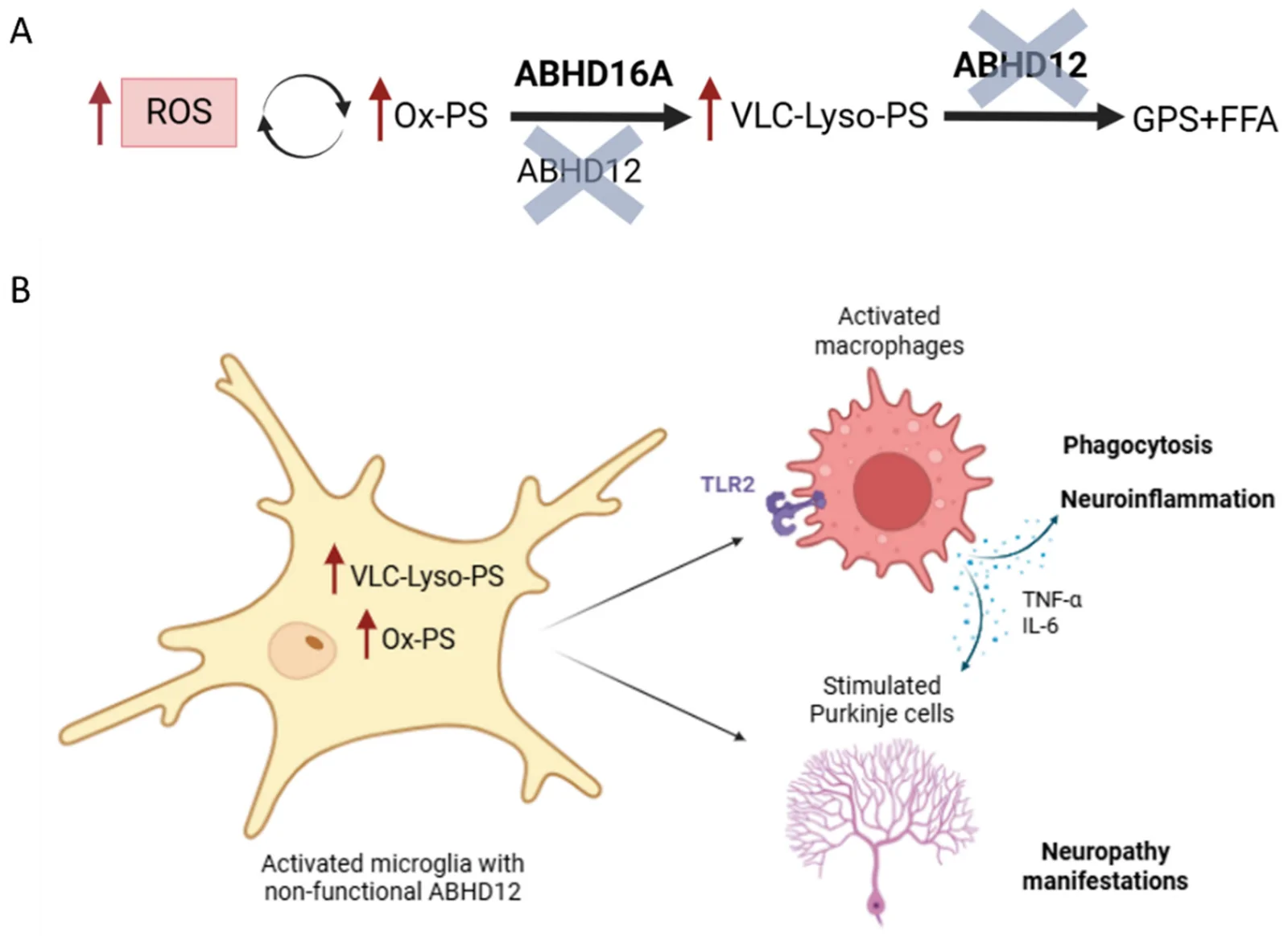
Proposed pathophysiological mechanism in PHARC syndrome. (**A**) Oxidized PS (ox-PS) generated under oxidative stress conditions is hydrolyzed by ABHD16A lipase, with the contribution of ABHD12, to lysophosphatidylserine (lysoPS), which is hydrolyzed by ABHD12 into glycerophosphoserine (GPS) and free fatty acid (FFA). (**B**) The presence of null mutations in the *ABHD12* gene causes loss of ABHD12 lipase activity, particularly in the microglia, where *ABHD12* expression is enriched. This results in the accumulation of both VLC-lysoPS and ox-PS lipids [7] in the brain, which could contribute to an increase in phagocytosis and neuroinflammation processes in the nervous system [16]. Secretion of proinflammatory cytokines (TNF-α and IL-6) is induced by VLC-lysoPS in macrophages via the TLR2 pathway [18]. Purkinje neurons are overstimulated with both VLC-lysoPS and proinflammatory cytokines, which could give rise to the neurobehavioral abnormalities associated with the PHARC neurological disorder [6,16,20].

Dysregulation of lipid metabolism in the central nervous system, followed by proinflammatory signalling linked to variants in *ABHD12*, has been proposed as a cause of the phenotypic features displayed in PHARC syndrome [1,7,15,16,17]. The accumulation of very-long-chain (VLC)-lyso-PS levels in the central nervous system in *Abhd12* knockout in vivo models has highlighted aberrant lipid metabolism as the central pathophysiological mechanism [6,15,18]. Additionally, the ABHD12 enzyme has recently been identified as a regulator of intracellular oxidized PS (ox-PS) levels in the mammalian brain [15,16]. Intracellularly, VLC-lyso-PS are mainly produced by the lipase ABHD16A [7], with additional contributions from ABHD12 through the hydrolysis of ox-PS molecules formed under oxidative stress conditions [15]. VLC-lyso-PS are then degraded by ABHD12 into glycerophosphoserine (GPS) and a free fatty acid (FFA) (Figure 1A). The presence of null mutations in *ABHD12* causes loss of ABHD12 lipase activity, particularly in the microglia, where ABHD12 expression is enriched. This results in the accumulation of both VLC-lyso-PS and ox-PS lipids in the brain [15], especially in the cerebellum, which is responsible for fine motor control, posture and balance, as observed in *Abhd12* knockout mouse models [6,19]. Both lipids trigger the secretion of proinflammatory cytokines (TNF-α and IL-6) in macrophages via the TLR2 pathway, which could contribute to an increase in phagocytosis and neuroinflammation processes in the nervous system [6]. Aberrant management of the phagocytosis mechanism has recently been identified as an important process in ABHD12 loss-related neuroinflammation, consistent with the role of lyso-PS and ox-PS in macrophage activation and inflammation [7,15,16,17]. In PHARC patients, an excess of VLC-lyso-PS and proinflammatory cytokines may constantly stimulate Purkinje neurons, and these uncontrolled events could ultimately lead to a neuropathy [1,6,7,16,20] (Figure 1B).

Here, we describe the clinical spectrum of 69 patients with *ABHD12* variants reported in the literature and diagnosed at Moorfields Eye Hospital (London, UK) to establish a genotype–phenotype correlation.

## 2. Results

### 2.1. Moorfields Eye Hospital Patients

**Figure 2:**
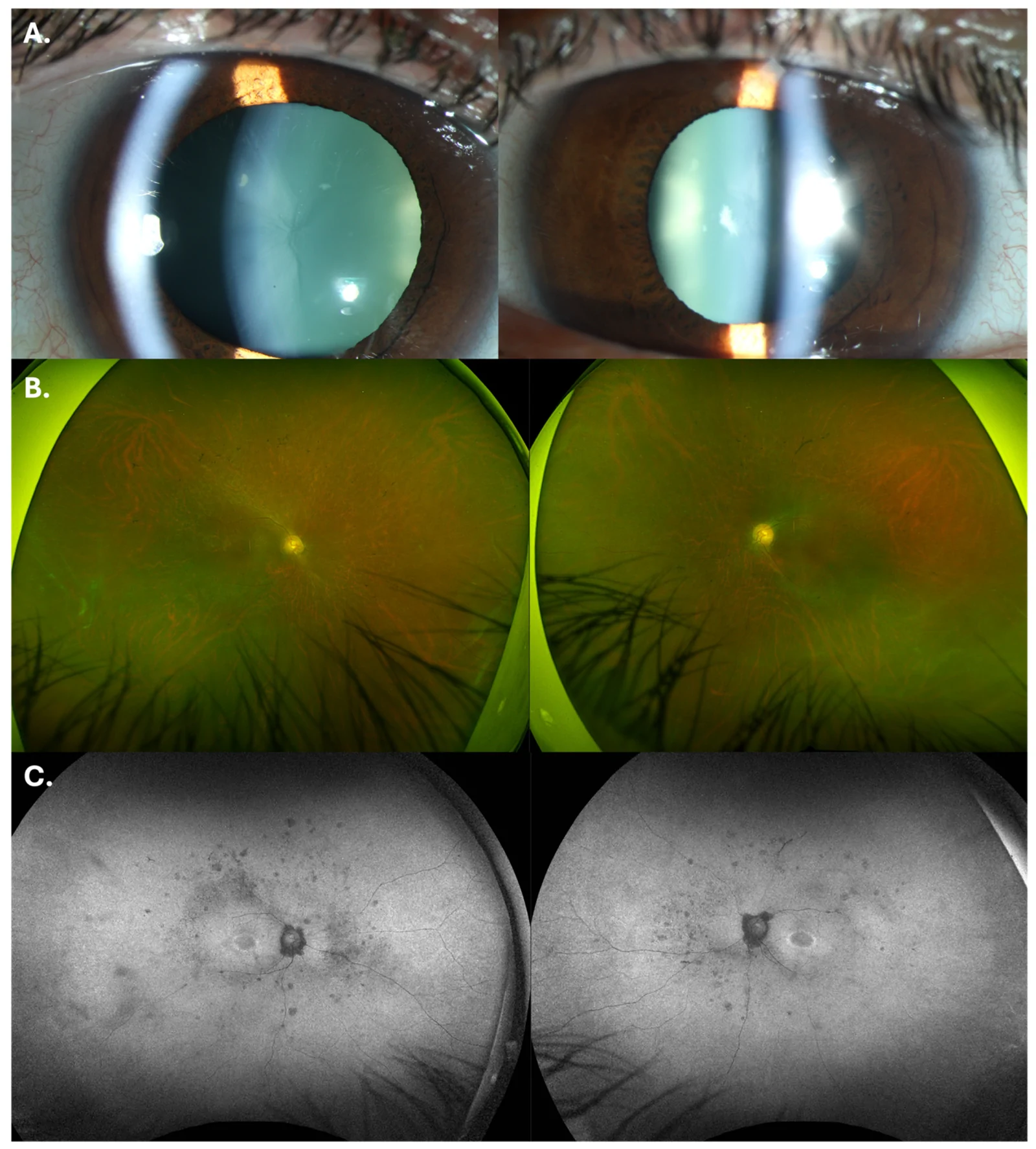
Clinical findings in a 48-year-old female patient with PHARC syndrome carrying a homozygous *ABHD12* c.193C>T (p.Arg65Ter) variant (patient 39.1). Best-corrected visual acuity was 6/12 and 6/15. (**A**) Anterior segment photography reveals a clear lens in the right eye and mild central lens opacity in the left eye. (**B**) Widefield color fundus images show attenuated retinal vessels and a waxy-pale optic disk but no bone spicules or pigment migration. (**C**) Fundus autofluorescence (FAF) imaging highlights areas of retinal degeneration, with hypo-autofluorescent patches corresponding to regions of photoreceptor loss.

A total of five patients from four unrelated families were included from Moorfields Eye Hospital (Table 1, 39.1, 39.2, 40.1, 41.1 and 42.1). Three patients carried a nonsense variant in homozygosity in exon 2 (c.193C>T, p.(Arg65*)), predicted to result in complete loss of ABHD12 function (Figure 2). Two of these patients are siblings and presented with all clinical features, except ataxia, which was absent in one of them. The third patient, aged 33 years, has developed only two features: RP, diagnosed at 20 years of age, and hearing loss, diagnosed at 18 years of age. A fourth patient was compound heterozygous for a nonsense variant and a splice site variant (c.784C>T/c.867+5G>A, p.(Arg262*)/p.(?)) in exon 8 and intron 9, respectively, and presented with all clinical features except cataracts. Lastly, we report a novel variant not previously described in the literature: c.985del p.(His329Thrfs*57), a frameshift variant in exon 11, predicted to lead to a shorter protein or absence of protein due to nonsense-mediated decay (NMD). This patient experienced all clinical features associated with PHARC syndrome, but had been diagnosed with Refsum disease prior to genetic testing.

### 2.2. Significant Clinical Variability in PHARC Patients

A total of 69 patients with PHARC syndrome were included in this study, including 5 patients from 4 unrelated families diagnosed at Moorfields Eye Hospital, London, UK, and 64 patients from 43 unrelated families from the literature review (Table 1) [1,2,3,21,22,23,24,25,26,27,28,29,30,31,32,33,34,35]. This comprises all patients with PHARC syndrome reported to date in whom genotypic and phenotypic information is provided.

**Table 1:** Clinical characteristics and *ABHD12* variants of PHARC patients. A total of 69 patients from 47 unrelated families have been genetically diagnosed with PHARC syndrome. To facilitate the identification of families, patients belonging to the same family share the first number of their ID. Age of onset of clinical features (where known) shown in brackets. P: polyneuropathy; H: hearing loss; A: ataxia; R: retinitis pigmentosa; C: cataract; ND: not documented. * Predicted with Missense3D version 2; ** predicted with SpliceAI (https://spliceailookup.broadinstitute.org/, accessed 4 August 2026).

Patient ID	*ABHD12* Variants	Protein Change	In Silico Prediction	Type of Mutation	P	H	A	R	C	Ref.
1.1	c.337_338delinsTTT	p.(Asp113Phefs*15)	-	Frameshift	Yes (30s)	Yes (20s)	No	Yes (38)	Yes (28)	[1]
1.2	c.337_338delinsTTT	p.(Asp113Phefs*15)	-	Frameshift	Yes (30s)	Yes (30s)	Yes (37)	Yes (37)	Yes (37)	[1]
1.3	c.337_338delinsTTT	p.(Asp113Phefs*15)	-	Frameshift	Yes (38)	Yes (Childhood)	Yes (43)	Yes (46)	Yes (25)	[1]
2.1	c.337_338delinsTTT	p.(Asp113Phefs*15)	-	Frameshift	Yes (51)	Yes (20s)	No	Yes (35)	Yes (26)	[1]
2.2	c.337_338delinsTTT	p.(Asp113Phefs*15)	-	Frameshift	Yes (53)	Yes (20s)	No	Yes (25)	Yes (25)	[1]
3.1	c.337_338delinsTTT	p.(Asp113Phefs*15)	-	Frameshift	Yes	Yes (10)	Yes	Yes (36)	Yes (32)	[1]
4.1	c.337_338delinsTTT	p.(Asp113Phefs*15)	-	Frameshift	Yes	Yes (10s)	No	No	Yes (15)	[1]
5.1	c.337_338delinsTTT	p.(Asp113Phefs*15)	-	Frameshift	Yes	Yes (13)	No	No	Yes (16)	[1]
6.1	g.25312257_25326263del14007insGG	Deletion of promoter region, exon 1	-	Deletion	Yes (Childhood)	Yes (14)	Yes	Yes (20s)	Yes (15)	[1]
6.2	g.25312257_25326263del14007insGG	Deletion of promoter region, exon 1	-	Deletion	Yes (Childhood)	Yes (6)	Yes (2)	Yes	Yes	[1]
6.3	g.25312257_25326263del14007insGG	Deletion of promoter region, exon 1	-	Deletion	Yes (Childhood)	Yes	Yes	No	Yes	[1]
7.1	c.1054C>T	p.(Arg352*)	-	Nonsense	Yes (34)	Yes (17)	Yes (18)	Yes (20s)	Yes (22)	[1]
8.1	c.846_852dupTAAGAGC	p.(His285*)	-	Nonsense	Yes	No	Yes (3)	No	No	[1]
8.2	c.846_852dupTAAGAGC	p.(His285*)	-	Nonsense	Yes	No	Yes (4)	No	No	[1]
9.1	c.846_852dupTAAGAGC	p.(His285*)	-	Nonsense	Yes	Yes	Yes (7)	No	ND	[1]
9.2	c.846_852dupTAAGAGC	p.(His285*)	-	Nonsense	Yes	Yes	Yes (4)	Yes	Yes	[1]
10.1	c.846_852dupTAAGAGC	p.(His285*)	-	Nonsense	Yes	Yes (6)	Yes (6)	No	No	[1]
10.2	c.846_852dupTAAGAGC	p.(His285*)	-	Nonsense	Yes (12)	ND	No	ND	ND	[1]
11.1	c.846_852dupTAAGAGC	p.(His285*)	-	Nonsense	Yes	Yes	Yes (16)	No	No	[1]
12.1	c.784C>T	p.(Arg262*)	-	Nonsense	Yes	Yes (9)	Yes	No	Yes (21)	[21]
12.2	c.784C>T	p.(Arg262*)	-	Nonsense	Yes	Yes (12)	Yes	Yes	Yes (25)	[21]
13.1	c.337_338delinsTTT/c.1075del	p.(Asp113Phefs*15)/p.(Val359Phefs*27)	-	Frameshift/Frameshift	Yes (8)	Yes (28)	Yes (8)	Yes (45)	Yes (36)	[22]
14.1	c.337_338delinsTTT	p.(Asp113Phefs*15)	-	Frameshift	Yes (Childhood)	Yes (17)	Yes (45)	Yes (32)	Yes (32)	[22]
15.1	c.337_338delinsTTT/c.423-1_425del	p.(Asp113Phefs*15)/p.(?)	-/Exon 4 skipping **	Frameshift/Splice site	Yes (27)	No	Yes (27)	Yes (14)	Yes (3)	[22]
15.2	c.337_338delinsTTT/c.423-1_425del	p.(Asp113Phefs*15)/p.(?)	-/Exon 4 skipping **	Frameshift/Splice site	Yes (Childhood)	Yes	Yes (27)	Yes (21)	Yes (3)	[22]
15.3	c.337_338delinsTTT/c.423-1_425del	p.(Asp113Phefs*15)/p.(?)	-/Exon 4 skipping **	Frameshift/Splice site	Yes (Childhood)	Yes (20)	Yes (31)	Yes	Yes (4)	[22]
16.1	c.337_338delinsTTT	p.(Asp113Phefs*15)	-	Frameshift	ND	Yes (12)	Yes	Yes (31)	Yes (32)	[22]
17.1	c.784C>T/c.867+5G>A	p.(Arg262*)/p.(?)	-/Exon 9 skipping **	Nonsense/Splice site	Yes (53)	Yes (20)	ND	Yes (18)	No	[22]
18.1	c.620-2A>G	p.(?)	Exon 7 skipping **	Splice site	Yes (31)	Yes (20)	ND	Yes (22)	Yes (26)	[22]
19.1	c.193C>T	p.(Arg65*)	-	Nonsense	Yes (7)	No	ND	Yes (16)	No	[22]
20.1	c.374C>T/c.1154T>C	p.(Thr125Met)/p.(Leu385Pro)	Both structurally damaging *	Missense/Missense	ND	Yes (44)	ND	Yes (30)	Yes (41)	[22]
21.1	c.337_338delinsTTT	p.(Asp113Phefs*15)	-	Frameshift	Yes (20)	Yes (16)	No	Yes (16)	Yes (17)	[22]
21.2	c.337_338delinsTTT	p.(Asp113Phefs*15)	-	Frameshift	Yes (18)	Yes (10)	No	Yes (10)	Yes (10)	[22]
22.1	c.1063C>T	p.(Arg355*)	-	Nonsense	Yes (47)	Yes	Yes	Yes	Yes	[22]
23.1	c.337_338delinsTTT/c.341dup	p.(Asp113Phefs*15)/p.(Leu114Phefs*14)	-	Frameshift/Frameshift	ND	Yes (33)	ND	Yes (23)	Yes (29)	[22]
24.1	c.319del/c.605C>T	p.(Arg107Glufs*8)/p.(Thr202Ile)	Structurally damaging *	Frameshift/Missense	No	Yes	No	Yes	Yes	[3]
24.2	c.319del/c.605C>T	p.(Arg107Glufs*8)/p.(Thr202Ile)	Structurally damaging *	Frameshift/Missense	No	Yes	Yes	Yes	Yes	[3]
24.3	c.319del/c.605C>T	p.(Arg107Glufs*8)/p.(Thr202Ile)	Structurally damaging *	Frameshift/Missense	No	No	Yes	Yes	Yes	[3]
24.4	c.319del/c.605C>T	p.(Arg107Glufs*8)/p.(Thr202Ile)	Structurally damaging *	Frameshift/Missense	No	Yes	Yes	Yes	Yes	[3]
25.1	c.477G>A/c.557G>C	p.(Trp159*)/p.(Arg186Pro)	-/Highly structurally damaging *	Nonsense/Missense	Yes (35)	Yes (36)	Yes	Yes (29)	Yes (29)	[3]
26.1	c.1116C>G	p.(His372Gln)	Structurally damaging *	Missense	Yes	Yes (37)	No	Yes	Yes	[3]
27.1	c.1054C>T/c.1196del	p.(Arg352*)/p.(*399Ser*122)	-	Nonsense/Frameshift	ND	ND	ND	Yes (18)	Yes (18)	[23]
28.1	c.1063C>T/c.259C>A	p.(Arg355*)/p.(Pro87Thr)	-/Benign *	Nonsense/Missense	ND	ND	ND	Yes (20)	Yes (20)	[23]
29.1	c.846_852dupTAAGAGC	p.(His285*)	-	Nonsense	ND	Yes (23)	ND	Yes (23)	Yes (23)	[24]
30.1	c.193C>T	p.(Arg65*)	-	Nonsense	ND	Yes (14)	Yes (19)	Yes (30s)	Yes (18)	[2]
30.2	c.193C>T	p.(Arg65*)	-	Nonsense	ND	Yes (14)	ND	Yes (23)	Yes (18)	[2]
31.1	c.211_223del	p.(Arg71Tyrfs*26)	-	Frameshift	Yes (11)	Yes (30)	Yes	Yes	Yes (38)	[25]
31.2	c.211_223del	p.(Arg71Tyrfs*26)	-	Frameshift	Yes (13)	Yes (20)	Yes	Yes	Yes	[25]
32.1	c.379_385delinsGATTCCTTATATACCATTGTAGTCTTACTGCTTTTGGTGAACACA	p.(Asn127Aspfs*23)	-	Frameshift	Yes (15)	Yes (5)	Yes (15)	No	Yes (28)	[26]
33.1	c.1129A>T/g.25338399_25340800del59000ins97bp	p.(Lys377*)/Deletion of promoter region, exon 1	-	Nonsense/Deletion	Yes	Yes (Childhood)	Yes	Yes	Yes (21)	[27]
34.1	c.249C>G	p.(Tyr83*)	-	Nonsense	No	Yes (33)	No	Yes (33)	Yes	[28]
34.2	c.249C>G	p.(Tyr83*)	-	Nonsense	No	Yes (32)	No	Yes (32)	Yes	[28]
35.1	c.758C>G	p.(Thr253Arg)	Structurally damaging *	Missense	Yes	Yes (15)	Yes	Yes	Yes (8)	[29]
36.1	c.316+2T>A	p.(?)/p.(?)	Partial intron 2 retention **	Splice site	Yes	Yes (Childhood)	No	Yes (45)	Yes (45)	[30]
36.2	c.316+2T>A	p.(?)/p.(?)	Partial intron 2 retention **	Splice site	No	Yes	No	Yes	No	[30]
37.1	c.316+2T>A	p.(?)/p.(?)	Partial intron 2 retention **	Splice site	Yes	Yes (15)	No	Yes (22)	Yes (18)	[30]
38.1	c.1054C>T	p.(Arg352*)	-	Nonsense	Yes (29)	Yes (16)	Yes (29)	Yes (19)	Yes (19)	[31]
39.1	c.193C>T	p.(Arg65*)	-	Nonsense	Yes (44)	Yes (13)	Yes	Yes (20)	Yes (42)	This study
39.2	c.193C>T	p.(Arg65*)	-	Nonsense	Yes (41)	Yes (10)	No	Yes (37)	Yes (41)	This study
40.1	c.985del	p.(His329Thrfs*57)	-	Frameshift	Yes	Yes (10)	Yes (10)	Yes (20)	Yes (53)	This study
41.1	c.193C>T	p.(Arg65*)	-	Nonsense	No	Yes (18)	No	Yes (20)	No	This study
42.1	c.784C>T/c.867+5G>A	p.(Arg262*)/p.(?)	-/Exon 9 skipping **	Nonsense/Splice site	Yes	Yes (19)	Yes	Yes (20)	No	This study
43.1	c.477G>A/g.25302218_25320318del	p.(Trp159*)/deletion of exons 4 to 12	-	Nonsense/Deletion	No	Yes (16)	Yes (Childhood)	Yes (Childhood)	Yes (Childhood)	[35]
44.1	c.690G>A	p.(Trp230*)	-	Nonsense	No	Yes (12)	Yes (24)	Yes (16)	Yes (22)	[34]
44.2	c.690G>A	p.(Trp230*)	-	Nonsense	No	Yes (8)	No	No	No	[34]
45.1	c.874C>T/c.205_206del	p.(Arg292*)/p.(Trp69Valfs*44)	-	Nonsense/Frameshift	No	Yes (13)	No	Yes (18)	No	[34]
46.1	c.871del	p.(Tyr291Ilefs*28)	-	Frameshift	Yes (23)	Yes (11)	Yes (23)	Yes (22)	Yes (14)	[33]
47.1	c.601dup	p.(Val201Glyfs*4)	-	Frameshift	Yes	Yes (11)	Yes	Yes	Yes	[32]
47.2	c.601dup	p.(Val201Glyfs*4)	-	Frameshift	Yes	Yes (11)	No	Yes	Yes	[32]

*ABHD12* variants and clinical characteristics of PHARC patients.

**Figure 3:**
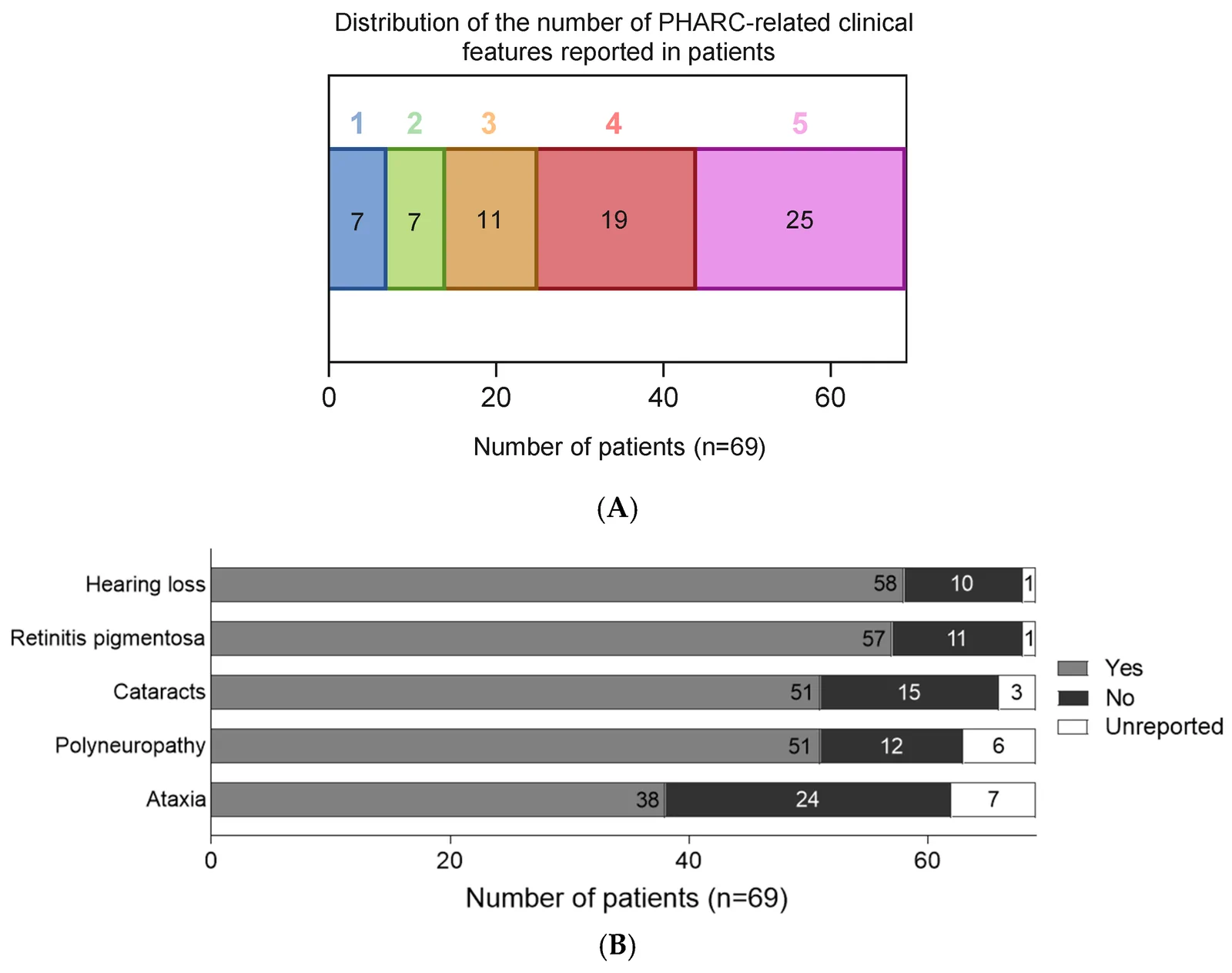
The significant clinical variability of PHARC syndrome. (**A**) Distribution of the number of PHARC-related clinical features (from 1 to 5) reported among 69 patients. Numbers within bars indicate absolute patient counts. (**B**) Number of patients that present each clinical feature. Hearing loss (58/69) is the most common clinical feature among patients with PHARC syndrome, closely followed by retinitis pigmentosa (57/69), cataracts (51/69) and polyneuropathy (51/69). Ataxia is the least common clinical manifestation (38/69).

In this cohort, there were 39 related patients (56.5%), a majority of males (59.4%), and almost a third of patients (31.8%) had a history of familial consanguinity. Of all the patients, 20.3% (14/69) reported one or two of the PHARC clinical features, whereas a majority of 63.8% (44/69) exhibited four or five features (Figure 3A). Considering that only half of the patients have their age reported in the literature, and that the age range of onset is quite wide, some of the patients exhibiting fewer clinical features may develop additional features later in life. Hearing loss and RP were the most frequently reported, present in 58 (84%) and 57 (82.6%), respectively (Figure 3B). However, these findings should be interpreted cautiously, as age and diagnostic data for some patients are incomplete or lack necessary assessments, such as neurological evaluations. Notably, ataxia and polyneuropathy were unreported in seven and six patients, respectively. Comprehensive phenotyping and long-term follow-up are essential for improving our understanding of the condition.

**Figure 4:**
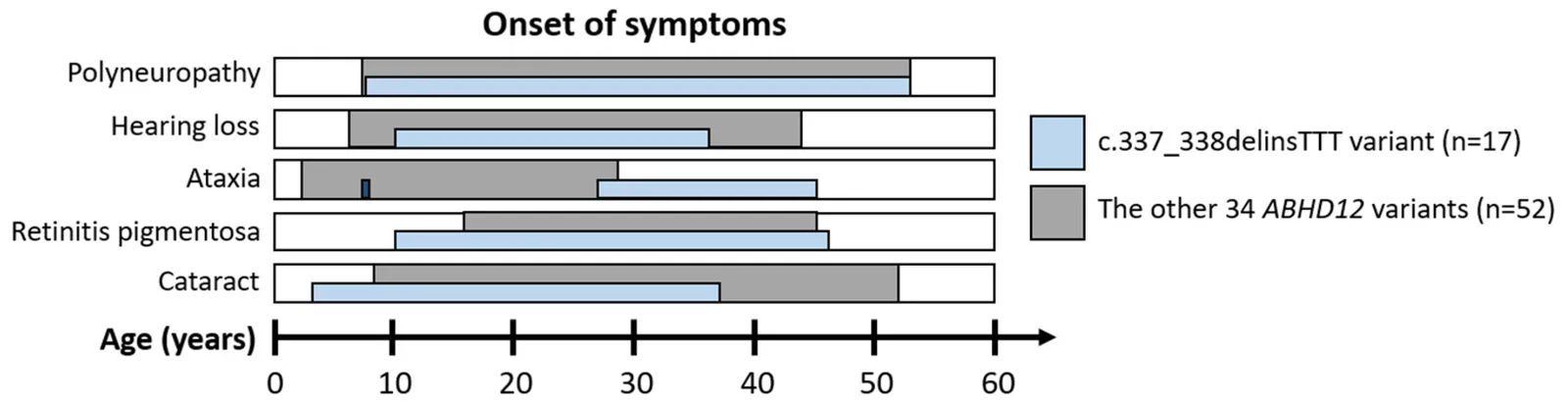
Age range of onset of each clinical feature in patients with *ABHD12* variants. In grey, the age of onset of each feature in patients with all *ABHD12* variants excluding the 17 patients harboring the most common variant (c.337_338delinsTTT), whose age range of onset is in blue. Among the five clinical features of PHARC syndrome, the age of onset of ataxia differs the most between c.337_338delinsTTT and the other 34 *ABHD12* variants. The only exception is a patient (c.337_338delinsTTT/c.1075del) whose ataxia symptoms started at age 8 years (dark blue bar).

PHARC syndrome demonstrates significant variability in the age of onset for its clinical features (Figure 4). Polyneuropathy symptoms have been observed as early as childhood, while some individuals are not diagnosed until the age of 53. Hearing loss, cataracts, and RP manifest between childhood and the fourth decade of life. Ataxia is the earliest diagnosed feature, with onset before 30 years of age, although its presence was only confirmed in around half of patients. An exception was observed in 17 patients of the cohort carrying the most prevalent variant (c.337_338delinsTTT, a frameshift in exon 3), either as homozygous or compound heterozygous, where ataxia onset ranged from the late-20s to mid-40s. One unique case involves a patient with a compound heterozygous genotype (c.337_338delinsTTT and c.1075del, a frameshift in exon 12) whose ataxia began at 8 years old (Figure 4, dark blue bar). The rest of her clinical features fit into the age range of c.337_338delinsTTT-carrying patients. It should be noted that age of symptom onset was reported only in a proportion of positive cases (86% for hearing loss, 82% for cataracts, 81% for RP, 60% for ataxia and 57% for polyneuropathy).

### 2.3. Common Variants and Large Deletions–Insertions

**Figure 5:**
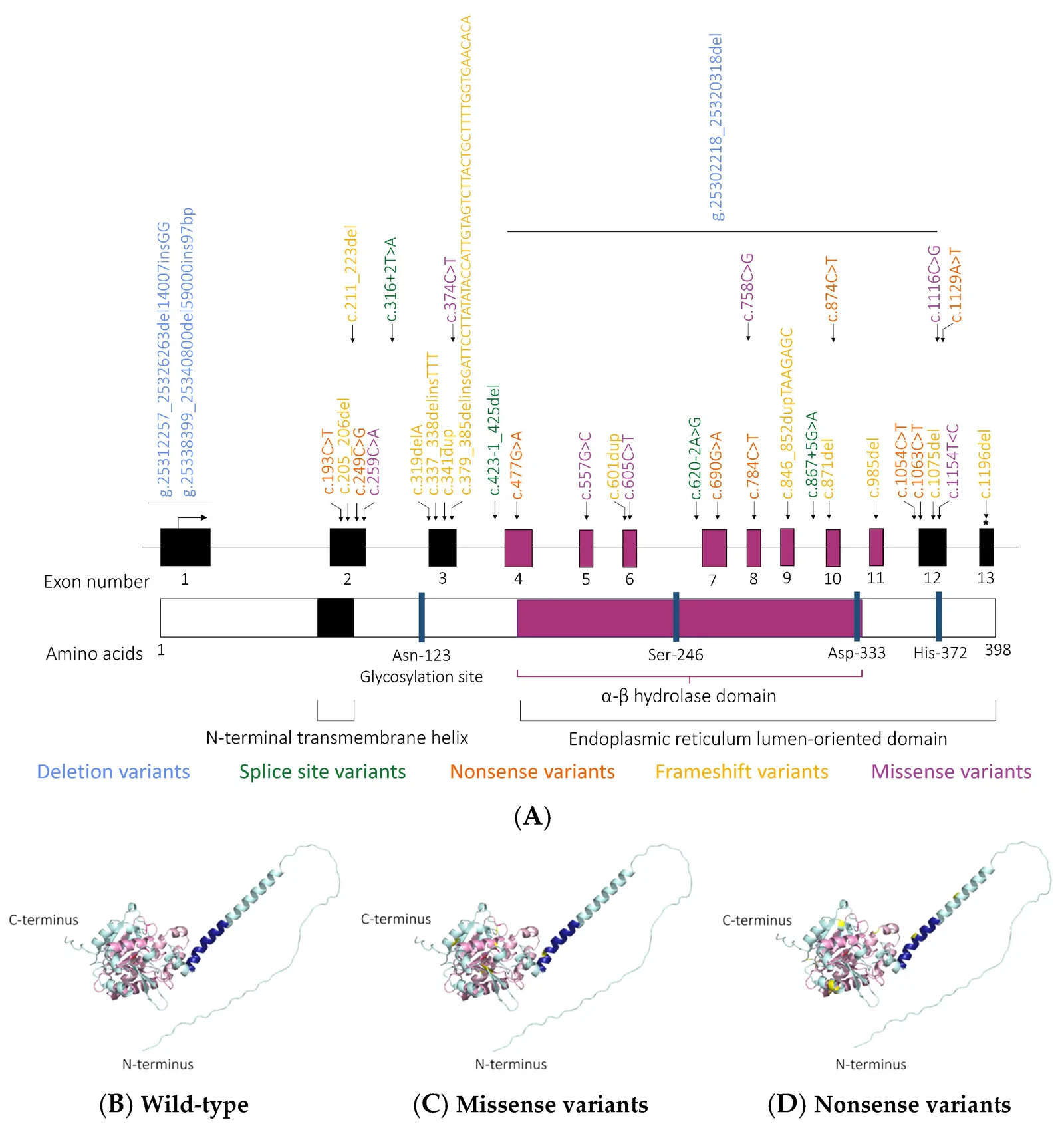
Mutational spectrum of *ABHD12*-related PHARC syndrome. (**A**) Variants are depicted across the 13 exons of the *ABHD12* gene, isoform a (NM_001042472.2). The α-β hydrolase domain [amino acid 171 to 335] is encoded from exon 4 to exon 11 and is shown in purple. The glycosylation site (amino acid Asn-123) and the three active sites (amino acids Ser-246, Asp-333 and His-372) are in blue (Uniprot Q8N2K0). * Translation termination codon. (**B**) ABHD12 protein structure predicted using AlphaFold and visualised using PyMOL (v2.5.8), showing α-β hydrolase domain (light pink); N-terminal transmembrane domain (blue); Asn-123 glycosylation site (green); and the three active sites (magenta). (**C**) Missense variants (yellow). (**D**) Nonsense variants (yellow).

A total of 35 unique variants in *ABHD12* (NM_001042472.2) have been identified in this cohort: 12 frameshift, 9 nonsense, 7 missense, 4 splice site, and 3 large deletion–insertion variants (Figure 5A). The variants are distributed across the whole gene; however, almost half of them (16/35) are located in exons 2, 3 or 12.

The most prevalent *ABHD12* variant in our cohort is c.337_338delinsTTT p.(Asp113Phefs*15), present in 17 patients. This variant was first described in an extended Norwegian family who were descendants of the same parents nine generations before [5]. This variant was identified in homozygosity in 12 patients from 8 unrelated families and in compound heterozygosity in 5 patients from 3 unrelated families, with c.1075del, c.423-1_425del and c.341dup as the second allele in one family each [1,22]. Almost all patients present with polyneuropathy (16/17), RP (15/17), cataracts (17/17), and hearing loss (16/17), regardless of the homozygous or heterozygous state. Ataxia, however, is only present in 9 out of the 17 patients. Interestingly, out of the five compound heterozygous patients, three siblings whose second allele was c.423-1_425del had particularly early-onset disease (Table 1, patients 15.1, 15.2, 15.3). All developed cataracts at 3–4 years old and RP during adolescence, and two developed polyneuropathy during childhood. The c.423-1_425del variant harbored by these siblings is a splice variant within intron 3, which could lead to several changes at the protein level (e.g., alternative splicing or truncated protein), making it difficult to predict the impact.

The second most frequently reported variant in our cohort is c.846_852dupTAAGAGC p.(His285fs*1), identified only in homozygosity in eight patients from five unrelated families [1,24]. These patients develop fewer clinical features than those carrying the c.337_338delinsTTT variant, with 75% (6/8) manifesting three features or less. Polyneuropathy (7/8), ataxia (7/8) and hearing loss (5/8) are most prevalent in this group, with ocular manifestations reported in only two patients.

Of the 35 disease-causing variants in *ABHD12* included in the cohort, two large deletions–insertions encompassing the promoter region and exon 1 (g.25312257_25326263del14007insGG; g.25338399_25340800del59000ins97bp) were identified in one family each [1,27]. Another large deletion expanding exons 4 to 12 of the *ABHD12* gene was identified in one patient [35]. The three variants are predicted to result in total absence of protein production. In our cohort, the first deletion–insertion variant (g.25312257_25326263del14007insGG, 14 kbp deletion) is present in three siblings in homozygosity (Table 1, patients 6.1, 6.2, 6.3). In the two older siblings (24 and 20 years old), there is a total penetrance of PHARC syndrome: they have developed polyneuropathy and ataxia in childhood, and hearing loss, RP and cataracts during childhood or adolescence. The youngest sibling (6 years old) presents all clinical features except for RP [1]. The second deletion–insertion variant (g.25338399_25340800del59000ins97bp, 59 kbp deletion) was present in a female patient (Table 1, patient 33.1) who also carried a nonsense variant in exon 12 (c.1129A>T, p.(Lys377*)). The deletion affects both the promoter and exon 1 of *ABHD12* and the promoter and exons 1–4 of *GINS1*. *GINS1* is involved in DNA replication, and its deficiency has been linked to pre- and post-natal growth retardation [36]. The patient (29 years old) presented with all five clinical features of PHARC [27].

### 2.4. Missense Variants Are Associated with Milder Phenotypes of PHARC Syndrome in This Cohort

**Figure 6:**
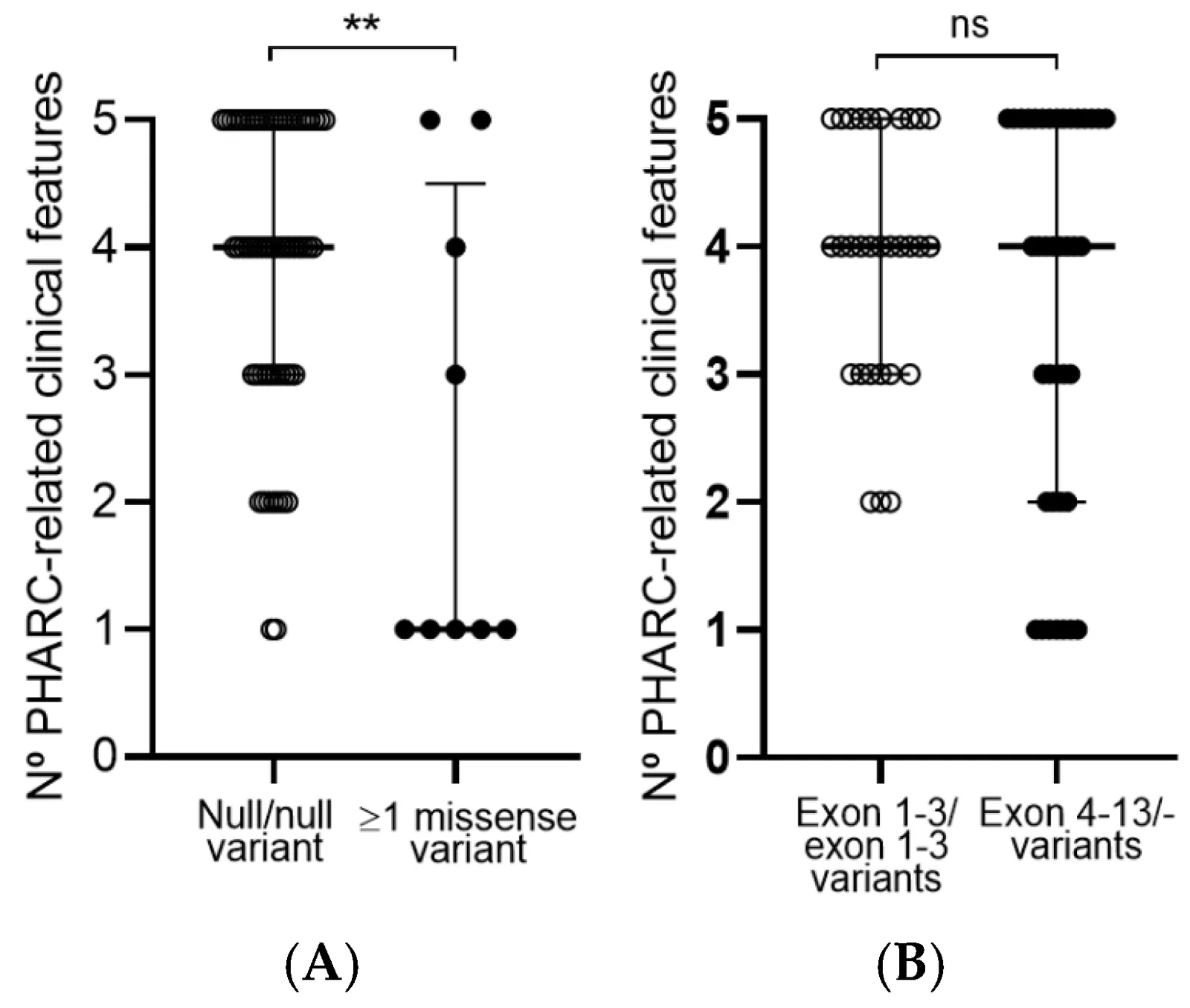
Analysis of the number of clinical features and the type and position of *ABHD12* variants in PHARC patients. (**A**) Significant statistical differences in terms of the number of clinical features displayed were found between patients carrying two null variants (n = 60) compared to patients carrying at least one missense variant in our dataset (n = 9) (*p* = 0.009). (**B**) No significant statistical differences in terms of the number of clinical features displayed were found between patients carrying both variants in exons 1 to 3 (inclusive) (n = 30) compared to patients carrying at least one mutation beyond exon 4 (n = 39). Results are expressed as median with interquartile range. Statistical analyses were performed by one-tailed Mann–Whitney test, ** *p* < 0.01.

The first hypothesis we tested was that carrying at least one missense variant, which may result in a conservative amino acid substitution, would be associated with fewer PHARC-related clinical features. As shown in Figure 6A, the number of clinical features observed in patients carrying null variants in both alleles (n = 60, Mdn = 4) was significantly higher than in those carrying at least one missense variant in our dataset (n = 9, Mdn = 1); U = 144, *p* = 0.0097, and r_rb_ = 0.47. Most patients with null variants in both alleles reported four or five PHARC clinical features (68%), whereas only 15% had one or two features (polyneuropathy present in half of them and hearing loss in the other half). On the contrary, most patients carrying at least one missense variant presented with only one clinical feature (55%), and that was exclusively RP.

The second hypothesis we tested was that variants, regardless of the type, in earlier exons would be expected to produce greater functional disruption of the ABHD12 protein. The catalytic triad and the α/β hydrolase domain are encoded by exons 4–11, and site-directed mutagenesis has demonstrated that these are the active sites catalysing lipid hydrolysis [37]. Therefore, patients with variants located before exon 4 were predicted to entail a more severe phenotype than patients with variants located from exon 4 to exon 13. However, the comparative analysis of patients carrying both variants between exons 1 and 3 (inclusive) (n = 30, Mdn = 4) versus patients harboring variants beyond exon 4 (n = 39, Mdn = 4) showed no significant difference in the number of clinical features displayed (Figure 6B); U = 527, *p* = 0.2334, and r_rb_ = 0.1.

## 3. Discussion

Our study combines the data from all 64 PHARC patients with both genotype and phenotype described in the literature with 5 new PHARC patients from Moorfields Eye hospital (London, UK). We report 35 unique variants in *ABHD12* associated with this syndrome, including one novel variant, c.985del p.(His329Thrfs*57). Although the total number of patients genetically and clinically diagnosed is limited, based on this 69-patient cohort, we show that carrying at least one missense variant is associated with fewer PHARC-related clinical features.

The most prevalent *ABHD12* variant in our cohort is c.337_338delinsTTT p.(Asp113Phefs*15), a frameshift variant in exon 3 present in homozygosity in 12 patients and in compound heterozygosity in 5 patients. Most of these patients present 4 or 5 clinical features (14/17), with ataxia being the least frequently reported feature. The age of onset of all the clinical manifestations is highly variable, ranging from childhood to the early fifties. The high phenotypic penetrance and early onset of certain clinical features observed in these patients may be explained by the predicted loss-of-function effect of this variant located in exon 3. Two nucleotides at positions 337 and 338 are deleted and replaced by three nucleotides (TTT), shifting the reading frame from amino acid 113 onwards and producing a PTC 15 residues downstream of the glycosylation site (Asn-123). The transcript of the c.337_338delinsTTT variant is predicted to be targeted by NMD, resulting in complete abrogation of protein production [38]. However, NMD efficiency is variable among patients, even among those with the same variant, as we previously showed in four choroideremia patients with the same nonsense UGA mutation [39].

The second most prevalent variant reported in the literature is c.846_852dupTAAGAGC p.(His285fs*1), a frameshift variant in exon 9 identified in homozygosity in eight patients. This 7-nucleotide duplication spanning positions 846–852 in exon 9 shifts the reading frame from residue 285, introducing a PTC immediately after. This transcript is a strong candidate for NMD, resulting in complete loss-of-function due to reduced or absent ABHD12 protein. For any transcripts that escape NMD, the resulting protein would be severely truncated, lacking the entire downstream C-terminal region of the protein. His-285 is a fully conserved residue in the ABHD12 structure and it has been proposed to interact closely with the lyso-PS substrate [12]. However, despite the predicted loss-of-function mechanism, most of the patients homozygous for this variant present three or fewer clinical features, with ocular manifestations present in only two of the eight patients. Nonetheless, six of these eight patients are younger than 30 years old, which may indicate an earlier stage of disease progression rather than a milder phenotype. Notably, seven out of the eight patients presented with ataxia, which is the least frequently reported feature among PHARC patients in general. This finding would benefit from further investigation in future cohorts to determine whether it represents an association with this specific variant.

The three large deletions identified in five patients represent the most complete loss-of-function mechanism in this cohort, and it is reflected in the high phenotypic penetrance and early onset of certain clinical features observed in these patients. Unlike frameshift or nonsense variants, which still produce a transcript or a truncated protein, deletion of the promoter and exon 1 removes the transcription start site and translation initiation codon (ATG) entirely. This means no transcript is produced at all, rather than an aberrant one being degraded or partially translated. The 18 kb deletion of exons 4 to 12 identified in heterozygosis in a 24-year-old patient presenting all PHARC clinical features except for polyneuropathy is also predicted to completely abrogate the functional domain of the protein.

Null variants generally introduce a PTC or disrupt the normal reading frame, frequently triggering NMD or producing a severely truncated, non-functional protein. On the other hand, missense variants result in a full-length protein with a single amino acid substitution. Depending on the specific residue affected, this may only partially compromise protein folding, stability, or catalytic efficiency, rather than eliminating the protein product altogether. Missense variants in *ABHD12* are carried by patients who report fewer PHARC clinical features in our cohort. A detailed review of the clinical features in this cohort reveals that, while there is a significant phenotypic variability among patients with the same genetic variant, patients carrying the missense variants represented in our dataset showed fewer recorded PHARC-related clinical features. Patients with biallelic null variants more frequently reported four or five clinical features, whereas those carrying at least one missense variant typically showed only one or two, and they were exclusively ocular manifestations. Although ABHD12 is expressed throughout the central nervous system (enriched in microglial cells and macrophages), the retina may be especially sensitive to its loss, which could explain its high vulnerability to *ABHD12* variants. A representative example of patients harboring one missense variant and reporting only one PHARC-related clinical feature was patients 24.1, 24.2, 24.3 and 24.4, whose ages range from 66 to 78 years old. They carry a frameshift variant in exon 3 (c.319delA p.(Arg107Glufs*8)) and a missense variant in exon 6 (c.605C>T p.(Thr202Ile)), and their only symptom of PHARC syndrome is RP. The PTC in exon 3 is expected to be NMD-sensitive, but if it escaped this mechanism, the resulting protein would be extremely short and likely non-functional. On the other hand, the missense variant substitutes a polar amino acid (Thr) with a hydrophobic one (Ile), within the α-β hydrolase domain. Although this change appears important, it may not markedly impair ABHD12 function, or at least not enough to cause hearing loss and neurological manifestations. This substitution was predicted to be structurally damaging by Missense3D, driven by two independent mechanisms: an expansion of internal cavity volume (+103.5 Å^3^, above the ≥70 Å^3^ threshold), and the loss of a buried hydrogen bond normally formed by the threonine side-chain hydroxyl group, which is not formed by isoleucine’s non-polar side chain. Additionally, this missense variant was functionally analyzed in a zebrafish PHARC model and demonstrated the importance of the high conservation of the protein functional domain [29]. A transient *abhd12* knockdown with morpholino oligonucleotides targeting either the exon 10-intron 10 boundary or the sequence flanking the translation initiation codon of *abhd12* recapitulated all PHARC clinical features. The results were morphants with mild, moderate and severe phenotypes exhibiting mobility impairment, abnormal cerebellar development, ataxia, microphthalmia, an absence of lens clarification, and a low number of mechanosensory hair cells in the inner ear and lateral line system. Co-injection of human *ABHD12* mRNA significantly rescued the phenotype; however, zebrafish co-injected with *ABHD12* mRNA harboring missense and nonsense variants (c.758C>G p.(Thr253Arg), c.1054C>T p.(Arg352*), and c.605C>T p.(Thr202Ile)) had no improvement in phenotypic features [29].

Another representative example would be patient 28.1 (23 years old), who only reported RP and carried a nonsense variant in exon 12 (c.1063C>T p.(Arg355*)) and a missense variant in exon 2 (c.259C>A p.(Pro87Thr)). The patient did not exhibit hearing loss or cataracts (ataxia and polyneuropathy were not assessed). The missense variant lies in the transmembrane domain of the protein (aa 75–92) [12], and the substitution was predicted to be benign by Missense3D, with no structural criteria triggered, suggesting minimal disruption to protein folding or stability. This contrasts with its current classification in ClinVar as a variant of uncertain significance (VUS), reflecting the limited clinical and functional evidence available for this specific substitution. This variant is exceptionally rare in the general population, with an allele frequency of 0.002664% in gnomAD, supporting its rarity and consistency with a potential pathogenic role in this autosomal recessive condition, despite the benign structural prediction. The paucity of PHARC features reported could be explained by the minimal disruption in the ABHD12 protein folding by the missense variant, or by the position of the PTC in exon 12, which may allow escape from NMD and result in production of an almost full-length protein. Alternatively, given the patient’s young age, it is possible that additional PHARC-associated features have simply not yet emerged, consistent with the progressive nature of the disease.

There are currently no specific therapies available for patients affected with PHARC syndrome. Treatment options are limited to symptomatic management, such as cataract surgery or cochlear implants. Given that the underlying aetiology of PHARC syndrome seems to be attributed to metabolic and immunological factors, targeting pathways such as VLC-lysoPS or oxidative stress with antioxidants could represent a promising strategy. A phase I study on the effects of orally administered N-acetylcysteine (NAC) in patients with the RP phenotype suggests that NAC has the potential to slow cone dysfunction by protecting cones from the high oxidative stress present in RP retinas. NAC is a potent antioxidant, already approved for other indications, that may be promising for improving visual function in PHARC patients with RP [40].

Beyond local symptomatic treatments, approaches aiming to correct disease-causing *ABHD12* variants could impact the entire syndrome spectrum. As 85.5% of patients are carrying at least one nonsense or frameshift variant leading to a PTC, translational readthrough-induced drugs (TRIDs) could be a beneficial therapeutic approach. The primary objective of TRIDs is to distinguish between a normal termination codon and a PTC in the mRNA that resists NMD; insert the correct amino acid; and reinstate the synthesis of a full-length functional protein, i.e., ABHD12 lipase [41,42]. Additionally, gene therapy holds great potential for treatment of inherited retinal disorders, and successful clinical trials have demonstrated the viability of gene therapy to slow down the progression of retinal diseases (e.g., voretigene neparvovec, targeting biallelic variants in *RPE65* causing Leber Congenital Amaurosis). Augmentation therapy, supplying a functional exogenous copy of the *ABHD12* gene and thereby restoring the normal degradation of VLC-lysoPS, would avoid the accumulation of proinflammatory lipids in the central nervous system microglia and presumably reverse the pathological mechanisms associated with PHARC syndrome.

This study has several limitations inherent to the source literature and the rare nature of PHARC syndrome, with only 69 patients reported to date. The level of clinical detail provided by the original publications is highly heterogeneous and often limited, particularly regarding age at onset of individual symptoms and basic demographic data. These findings must therefore be interpreted cautiously given the incomplete clinical data available for some patients and the limited overall sample size, as well as the possibility that some individuals in our cohort are in a pre-clinical stage of the disease. This cannot be formally excluded, since PHARC is a progressive, multisystemic disorder and features are known to have variable and sometimes late age of onset. Additionally, whole-exome sequencing and targeted panel testing may fail to detect copy number variants, structural variants or single nucleotide variants and deletions–insertions located in non-coding regions such as the promoter. The phenotypic variability shown in this cohort of PHARC patients highlights the importance of a coordinated, interdisciplinary approach involving neurologists, ophthalmologists, audiologists, and geneticists to accurately diagnose PHARC syndrome and distinguish it from conditions with overlapping characteristics. Such collaboration is essential not only for optimal patient care, but also to facilitate the inclusion of patients in ongoing research studies that may advance our understanding of this complex disorder.

## 4. Materials and Methods

An extensive literature review was conducted up to July 2025. Using the terms “ABHD12” and “PHARC” in PubMed to collect all the relevant information related to patients genetically diagnosed with biallelic mutations in the *ABHD12* gene, 18 articles were included [1,2,3,21,22,23,24,25,26,27,28,29,30,31,32,33,34,35]; duplicated cases and cases without genetic information were excluded. Additionally, we report 5 patients from 4 unrelated families that were genetically diagnosed at Moorfields Eye Hospital NHS Foundation Trust, London, UK. Either whole-exome sequencing or targeted retinal gene panel testing was performed in the probands. For each patient, their sex, genetic variant and information about their clinical features were collected. Statistical analysis was performed using GraphPad Prism 8.0 (GraphPad Software Inc., San Diego, CA, USA). One-tailed Mann–Whitney test was used for comparisons, with significance achieved with *p* value of ≤0.05 (*). Reported U values correspond to the smaller of the two complementary Mann–Whitney U statistics. All results are expressed as median with interquartile range.

This study had relevant local and national research ethics committee approvals (Moorfields Eye Hospital NHS Foundation Trust and the Northwest London Research Ethics Committee) and adhered to the tenets of the Declaration of Helsinki. Patients and relatives gave written informed consent for genetic testing through either the Genetic Study of Inherited Eye Disease (REC reference 12/LO/0141) or the Genomics England 100,000 Genomes Project (REC reference 14/EE/1112).

## 5. Conclusions

We reported a novel frameshift variant, *ABHD12*: c.985del p.(His329Thrfs*57), in one patient reporting all five PHARC clinical features. The analysis of the phenotype and variants presented in this cohort has shown that patients with two null variants in *ABHD12* manifest more of the clinical features of PHARC syndrome than those with at least one missense variant. Furthermore, patients with the c.337_338delinsTTT p.(Asp113Phefs*15) *ABHD12* variant seem to develop ataxia at a later age than those with other variants. However, as PHARC syndrome is a rare disease and the number of cases reported worldwide remains small, further work needs to be undertaken to establish whether these conclusions remain true with a larger cohort. PHARC syndrome is often misdiagnosed or undiagnosed, leading to delayed or inadequate management for affected individuals. Identifying the pathophysiological mechanisms underlying this debilitating condition, raising awareness about the disorder among healthcare professionals and increasing genetic testing can improve the accuracy of diagnosis and treatment for those affected by PHARC syndrome.

## Data Availability

The original contributions presented in this study are included in the article. Further inquiries can be directed to the corresponding author.

## Abbreviations

The following abbreviations are used in this manuscript:

ABHD12	α/β-hydrolase domain-containing protein 12
NMD	Nonsense-mediated decay
PHARC	Polyneuropathy, hearing loss, ataxia, retinitis pigmentosa and cataracts
PTC	Premature stop codon
RP	Retinitis pigmentosa
VLC-lyso-PS	Very-long-chain lysophosphatidylserines
